# 2,4-Dichloro-*N*-(2,4-dimethyl­phen­yl)benzene­sulfonamide

**DOI:** 10.1107/S1600536811039274

**Published:** 2011-10-05

**Authors:** Vinola Z. Rodrigues, Sabine Foro, B. Thimme Gowda

**Affiliations:** a Department of Chemistry, Mangalore University, Mangalagangotri 574 199, Mangalore, India; b Institute of Materials Science, Darmstadt University of Technology, Petersenstrasse 23, D-64287 Darmstadt, Germany

## Abstract

In the title compound, C_14_H_13_Cl_2_NO_2_S, the C—SO_2_—NH—C torsion angle is −71.4 (4)°. The sulfonyl and aniline benzene rings are tilted relative to one another by 44.6 (1)°. The crystal structure features inversion-related dimers linked by pairs of N—H⋯O hydrogen bonds.

## Related literature

For the preparation of the title compound, see: Savitha & Gowda (2006 ▶). For hydrogen-bonding modes of sulfonamides, see: Adsmond & Grant (2001 ▶). For our studies on the effects of substituents on the structures and other aspects of *N*-(ar­yl)-amides, see: Arjunan *et al.* (2004 ▶); Gowda *et al.* (2000 ▶), on *N*-(ar­yl)-methane­sulfonamides, see: Gowda *et al.* (2007 ▶) and on *N*-(ar­yl)-aryl­sulfonamides, see: Gelbrich *et al.* (2007 ▶); Perlovich *et al.* (2006 ▶); Gowda *et al.* (2005 ▶); Rodrigues *et al.* (2011 ▶).
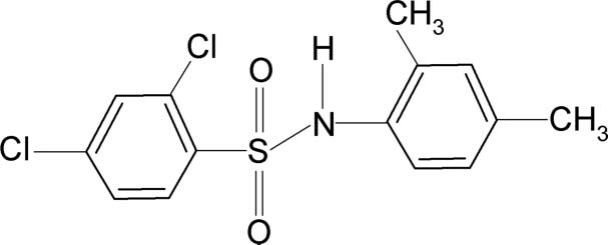


## Experimental

### 

#### Crystal data

C_14_H_13_Cl_2_NO_2_S
*M* *_r_* = 330.21
Monoclinic, 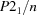

*a* = 8.2127 (7) Å
*b* = 12.619 (1) Å
*c* = 14.284 (1) Å
β = 90.434 (8)°
*V* = 1480.3 (2) Å^3^
*Z* = 4
Mo *K*α radiation
μ = 0.58 mm^−1^
*T* = 293 K
0.28 × 0.18 × 0.04 mm

#### Data collection

Oxford Diffraction Xcalibur diffractometer with a Sapphire CCD detector
Absorption correction: multi-scan (*CrysAlis RED*; Oxford Diffraction, 2009 ▶) *T*_min_ = 0.855, *T*_max_ = 0.977
5678 measured reflections
3007 independent reflections
1908 reflections with *I* > 2σ(*I*)
*R*_int_ = 0.038

#### Refinement

*R*[*F* ^2^ > 2σ(*F* ^2^)] = 0.075
*wR*(*F* ^2^) = 0.143
*S* = 1.20
3007 reflections
186 parameters
1 restraint
H atoms treated by a mixture of independent and constrained refinement
Δρ_max_ = 0.40 e Å^−3^
Δρ_min_ = −0.41 e Å^−3^

### 

Data collection: *CrysAlis CCD* (Oxford Diffraction, 2009 ▶); cell refinement: *CrysAlis RED* (Oxford Diffraction, 2009 ▶); data reduction: *CrysAlis RED*; program(s) used to solve structure: *SHELXS97* (Sheldrick, 2008 ▶); program(s) used to refine structure: *SHELXL97* (Sheldrick, 2008 ▶); molecular graphics: *PLATON* (Spek, 2009 ▶); software used to prepare material for publication: *SHELXL97*.

## Supplementary Material

Crystal structure: contains datablock(s) I, global. DOI: 10.1107/S1600536811039274/ds2145sup1.cif

Structure factors: contains datablock(s) I. DOI: 10.1107/S1600536811039274/ds2145Isup2.hkl

Supplementary material file. DOI: 10.1107/S1600536811039274/ds2145Isup3.cml

Additional supplementary materials:  crystallographic information; 3D view; checkCIF report

## Figures and Tables

**Table 1:** Hydrogen-bond geometry (Å, °)

*D*—H⋯*A*	*D*—H	H⋯*A*	*D*⋯*A*	*D*—H⋯*A*
N1—H1*N*⋯O2^i^	0.86 (2)	2.23 (2)	3.059 (5)	164 (4)

Symmetry code: (i) 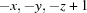.

**Fig. 1.**
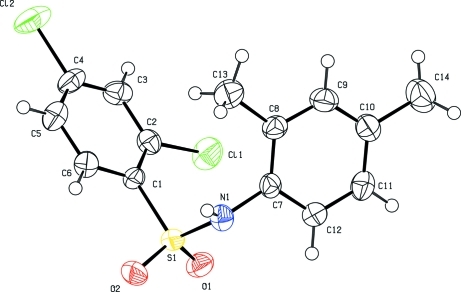
Molecular structure of the title compound, showing the atom labelling scheme and displacement ellipsoids are drawn at the 50% probability level.

**Fig. 2.**
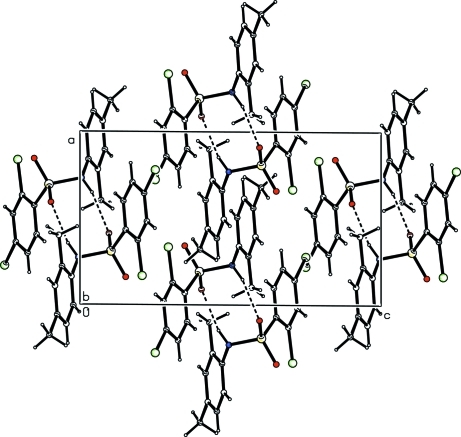
Molecular packing of (I) with hydrogen bonding shown as dashed lines.

## supplementary crystallographic information

### Comment

Several biologically important compounds contain the sulfonamide moiety. The hydrogen bonding preferences of sulfonamides have been investigated (Adsmond & Grant, 2001). As part of our studies on the substituent effects on the structures and other aspects of *N*-(aryl)-amides (Arjunan *et al.*, 2004; Gowda *et al.*, 2000), *N*-(aryl)-methanesulfonamides (Gowda *et al.*, 2007) and *N*-(aryl)-arylsulfonamides (Gowda *et al.*, 2005; Rodrigues *et al.*, 2011), in the present work, the crystal structure of 2,4-dichloro-*N*-(2,4-dimethylphenyl)-benzenesulfonamide (I) has been determined (Fig. 1).

The molecule is bent at the S atom with C—SO_2_—NH—C torsion angle of -71.38 (39)°, compared to the value of 67.45 (17)° in 4-chloro-2-methyl-N-(2,4-dimethylphenyl)benzenesulfonamide (II) (Rodrigues *et al.*, 2011). The sulfonyl and the aniline benzene rings are tilted relative to each other by 44.6 (1)°, compared to the value of 44.5 (1)° in (II).

The other bond parameters in (I) are similar to those observed in (II) and other aryl sulfonamides (Perlovich *et al.*, 2006; Gelbrich *et al.*, 2007).

In the crystal structure, the pairs of intermolecular N–H···O hydrogen bonds (Table 1) link the molecules into inversion-related dimers. Part of the crystal structure is shown in Fig. 2.

### Experimental

The solution of 1,3-dichlorobenzene (10 ml) in chloroform (40 ml) was treated dropwise with chlorosulfonic acid (25 ml) at 0 ° C. After the initial evolution of hydrogen chloride subsided, the reaction mixture was brought to room temperature and poured into crushed ice in a beaker. The chloroform layer was separated, washed with cold water and allowed to evaporate slowly. The residual 2,4-dichlorobenzenesulfonylchloride was treated with 2,4-dimethylaniline in the stoichiometric ratio and boiled for ten minutes. The reaction mixture was then cooled to room temperature and added to ice cold water (100 ml). The resultant solid 2,4-dichloro-*N*- (2,4-dimethylphenyl)-benzenesulfonamide was filtered under suction and washed thoroughly with cold water. It was then recrystallized to constant melting point from dilute ethanol. The purity of the compound was checked and characterized by recording its infrared and NMR spectra (Savitha & Gowda, 2006).

Plate like light pink single crystals used in X-ray diffraction studies were grown in ethanolic solution by slow evaporation at room temperature.

### Refinement

The H atoms of the NH groups were located in a difference map and later restrained to N—H = 0.86 (2) %A. The other H atoms were positioned with idealized geometry using a riding model with the aromatic C—H = 0.93Å and methyl C—H = 0.96 Å. All H atoms were refined with isotropic displacement parameters. The *U*_iso_(H) values were set at 1.2*U*_eq_(C-aromatic, N) and 1.5*U*_eq_(*C*-methyl).

### Crystal data

**Table:**

C_14_H_13_Cl_2_NO_2_S	*F*(000) = 680
*M**_r_* = 330.21	*D*_x_ = 1.482 Mg m^−^^3^
Monoclinic, *P*2_1_/*n*	Mo *K*α radiation, λ = 0.71073 Å
Hall symbol: -P 2yn	Cell parameters from 1307 reflections
*a* = 8.2127 (7) Å	θ = 2.9–28.1°
*b* = 12.619 (1) Å	µ = 0.58 mm^−^^1^
*c* = 14.284 (1) Å	*T* = 293 K
β = 90.434 (8)°	Plate, light pink
*V* = 1480.3 (2) Å^3^	0.28 × 0.18 × 0.04 mm
*Z* = 4	

### Data collection

**Table:**

Oxford Diffraction Xcalibur diffractometer with a Sapphire CCD detector	3007 independent reflections
Radiation source: fine-focus sealed tube	1908 reflections with *I* > 2σ(*I*)
graphite	*R*_int_ = 0.038
Rotation method data acquisition using ω scans	θ_max_ = 26.4°, θ_min_ = 2.9°
Absorption correction: multi-scan (*CrysAlis RED*; Oxford Diffraction, 2009)	*h* = −9→10
*T*_min_ = 0.855, *T*_max_ = 0.977	*k* = −15→15
5678 measured reflections	*l* = −17→11

### Refinement

**Table:**

Refinement on *F*^2^	Primary atom site location: structure-invariant direct methods
Least-squares matrix: full	Secondary atom site location: difference Fourier map
*R*[*F*^2^ > 2σ(*F*^2^)] = 0.075	Hydrogen site location: inferred from neighbouring sites
*wR*(*F*^2^) = 0.143	H atoms treated by a mixture of independent and constrained refinement
*S* = 1.20	*w* = 1/[σ^2^(*F*_o_^2^) + (0.0245*P*)^2^ + 3.0449*P*] where *P* = (*F*_o_^2^ + 2*F*_c_^2^)/3
3007 reflections	(Δ/σ)_max_ < 0.001
186 parameters	Δρ_max_ = 0.40 e Å^−^^3^
1 restraint	Δρ_min_ = −0.41 e Å^−^^3^

### Special details

**Table:**

Geometry. All e.s.d.'s (except the e.s.d. in the dihedral angle between two l.s. planes) are estimated using the full covariance matrix. The cell e.s.d.'s are taken into account individually in the estimation of e.s.d.'s in distances, angles and torsion angles; correlations between e.s.d.'s in cell parameters are only used when they are defined by crystal symmetry. An approximate (isotropic) treatment of cell e.s.d.'s is used for estimating e.s.d.'s involving l.s. planes.
Refinement. Refinement of *F*^2^ against ALL reflections. The weighted *R*-factor *wR* and goodness of fit *S* are based on *F*^2^, conventional *R*-factors *R* are based on *F*, with *F* set to zero for negative *F*^2^. The threshold expression of *F*^2^ > σ(*F*^2^) is used only for calculating *R*-factors(gt) *etc*. and is not relevant to the choice of reflections for refinement. *R*-factors based on *F*^2^ are statistically about twice as large as those based on *F*, and *R*- factors based on ALL data will be even larger.

### Fractional atomic coordinates and isotropic or equivalent isotropic displacement parameters (Å^2^)

**Table:**

	*x*	*y*	*z*	*U*_iso_*/*U*_eq_	
C1	0.0688 (5)	0.1655 (3)	0.3481 (3)	0.0296 (10)	
C2	0.1271 (5)	0.2584 (4)	0.3079 (3)	0.0343 (11)	
C3	0.0195 (6)	0.3363 (4)	0.2776 (3)	0.0394 (12)	
H3	0.0583	0.3987	0.2513	0.047*	
C4	−0.1440 (6)	0.3202 (4)	0.2870 (3)	0.0405 (12)	
C5	−0.2059 (6)	0.2293 (4)	0.3258 (3)	0.0411 (12)	
H5	−0.3177	0.2198	0.3315	0.049*	
C6	−0.0994 (5)	0.1527 (4)	0.3560 (3)	0.0358 (11)	
H6	−0.1401	0.0908	0.3824	0.043*	
C7	0.3390 (5)	0.1826 (4)	0.5282 (3)	0.0315 (10)	
C8	0.2812 (5)	0.2800 (4)	0.5595 (3)	0.0353 (11)	
C9	0.3947 (6)	0.3572 (4)	0.5828 (3)	0.0430 (12)	
H9	0.3573	0.4223	0.6043	0.052*	
C10	0.5609 (6)	0.3417 (4)	0.5755 (3)	0.0405 (12)	
C11	0.6145 (6)	0.2437 (4)	0.5452 (3)	0.0422 (12)	
H11	0.7257	0.2309	0.5404	0.051*	
C12	0.5057 (6)	0.1650 (4)	0.5221 (3)	0.0394 (12)	
H12	0.5439	0.0994	0.5022	0.047*	
C13	0.1017 (6)	0.3029 (5)	0.5700 (4)	0.0566 (15)	
H13A	0.0528	0.2493	0.6083	0.068*	
H13B	0.0504	0.3032	0.5094	0.068*	
H13C	0.0878	0.3709	0.5990	0.068*	
C14	0.6799 (7)	0.4288 (4)	0.6014 (4)	0.0625 (16)	
H14A	0.7536	0.4036	0.6488	0.075*	
H14B	0.6212	0.4890	0.6247	0.075*	
H14C	0.7403	0.4492	0.5470	0.075*	
N1	0.2303 (5)	0.0970 (3)	0.5054 (3)	0.0353 (9)	
H1N	0.147 (4)	0.086 (4)	0.540 (3)	0.042*	
O1	0.3411 (4)	0.0563 (3)	0.3481 (2)	0.0431 (8)	
O2	0.0892 (4)	−0.0289 (2)	0.4037 (2)	0.0439 (9)	
Cl1	0.33286 (15)	0.28541 (11)	0.29353 (11)	0.0557 (4)	
Cl2	−0.27868 (19)	0.41792 (12)	0.24989 (10)	0.0642 (5)	
S1	0.19166 (14)	0.06286 (9)	0.39726 (8)	0.0333 (3)	

### Atomic displacement parameters (Å^2^)

**Table:**

	*U*^11^	*U*^22^	*U*^33^	*U*^12^	*U*^13^	*U*^23^
C1	0.032 (2)	0.028 (2)	0.028 (2)	0.005 (2)	−0.0061 (19)	0.0004 (19)
C2	0.036 (3)	0.036 (3)	0.032 (3)	−0.003 (2)	0.002 (2)	−0.003 (2)
C3	0.055 (3)	0.031 (3)	0.032 (3)	0.004 (2)	0.000 (2)	0.006 (2)
C4	0.043 (3)	0.048 (3)	0.031 (3)	0.019 (2)	−0.004 (2)	0.001 (2)
C5	0.033 (3)	0.054 (3)	0.037 (3)	0.005 (2)	0.000 (2)	0.004 (2)
C6	0.035 (3)	0.037 (3)	0.035 (3)	−0.003 (2)	−0.001 (2)	0.005 (2)
C7	0.035 (2)	0.033 (3)	0.026 (2)	−0.002 (2)	−0.0066 (19)	0.003 (2)
C8	0.037 (3)	0.038 (3)	0.031 (3)	0.007 (2)	−0.001 (2)	−0.002 (2)
C9	0.050 (3)	0.036 (3)	0.043 (3)	0.006 (2)	0.000 (2)	−0.006 (2)
C10	0.048 (3)	0.036 (3)	0.037 (3)	−0.006 (2)	−0.008 (2)	0.004 (2)
C11	0.030 (2)	0.053 (3)	0.044 (3)	0.000 (2)	−0.002 (2)	0.000 (3)
C12	0.038 (3)	0.036 (3)	0.043 (3)	0.005 (2)	−0.006 (2)	−0.004 (2)
C13	0.043 (3)	0.062 (4)	0.065 (4)	0.010 (3)	0.002 (3)	−0.015 (3)
C14	0.065 (4)	0.052 (4)	0.070 (4)	−0.016 (3)	−0.010 (3)	0.002 (3)
N1	0.035 (2)	0.037 (2)	0.034 (2)	−0.0042 (19)	−0.0019 (17)	0.0029 (18)
O1	0.0390 (19)	0.044 (2)	0.046 (2)	0.0111 (17)	−0.0005 (15)	−0.0042 (17)
O2	0.051 (2)	0.0267 (18)	0.054 (2)	−0.0046 (16)	−0.0099 (17)	0.0003 (15)
Cl1	0.0384 (7)	0.0538 (9)	0.0749 (10)	−0.0073 (6)	0.0043 (6)	0.0165 (7)
Cl2	0.0728 (10)	0.0639 (10)	0.0556 (9)	0.0386 (8)	−0.0043 (7)	0.0067 (7)
S1	0.0363 (6)	0.0271 (6)	0.0365 (7)	0.0036 (5)	−0.0052 (5)	−0.0008 (5)

### Geometric parameters (Å, °)

**Table:**

C1—C2	1.392 (6)	C9—C10	1.383 (6)
C1—C6	1.396 (6)	C9—H9	0.9300
C1—S1	1.783 (4)	C10—C11	1.384 (7)
C2—C3	1.389 (6)	C10—C14	1.515 (7)
C2—Cl1	1.737 (4)	C11—C12	1.375 (6)
C3—C4	1.365 (6)	C11—H11	0.9300
C3—H3	0.9300	C12—H12	0.9300
C4—C5	1.374 (7)	C13—H13A	0.9600
C4—Cl2	1.737 (5)	C13—H13B	0.9600
C5—C6	1.370 (6)	C13—H13C	0.9600
C5—H5	0.9300	C14—H14A	0.9600
C6—H6	0.9300	C14—H14B	0.9600
C7—C12	1.391 (6)	C14—H14C	0.9600
C7—C8	1.392 (6)	N1—S1	1.632 (4)
C7—N1	1.437 (6)	N1—H1N	0.855 (19)
C8—C9	1.387 (6)	O1—S1	1.421 (3)
C8—C13	1.511 (6)	O2—S1	1.435 (3)
			
C2—C1—C6	118.4 (4)	C11—C10—C14	121.2 (5)
C2—C1—S1	125.4 (3)	C12—C11—C10	120.9 (4)
C6—C1—S1	116.2 (3)	C12—C11—H11	119.6
C3—C2—C1	120.3 (4)	C10—C11—H11	119.6
C3—C2—Cl1	116.2 (4)	C11—C12—C7	120.5 (4)
C1—C2—Cl1	123.5 (4)	C11—C12—H12	119.7
C4—C3—C2	119.2 (4)	C7—C12—H12	119.7
C4—C3—H3	120.4	C8—C13—H13A	109.5
C2—C3—H3	120.4	C8—C13—H13B	109.5
C3—C4—C5	122.1 (4)	H13A—C13—H13B	109.5
C3—C4—Cl2	119.3 (4)	C8—C13—H13C	109.5
C5—C4—Cl2	118.7 (4)	H13A—C13—H13C	109.5
C6—C5—C4	118.6 (4)	H13B—C13—H13C	109.5
C6—C5—H5	120.7	C10—C14—H14A	109.5
C4—C5—H5	120.7	C10—C14—H14B	109.5
C5—C6—C1	121.5 (4)	H14A—C14—H14B	109.5
C5—C6—H6	119.3	C10—C14—H14C	109.5
C1—C6—H6	119.3	H14A—C14—H14C	109.5
C12—C7—C8	120.0 (4)	H14B—C14—H14C	109.5
C12—C7—N1	118.4 (4)	C7—N1—S1	121.9 (3)
C8—C7—N1	121.6 (4)	C7—N1—H1N	119 (3)
C9—C8—C7	117.9 (4)	S1—N1—H1N	110 (3)
C9—C8—C13	119.7 (4)	O1—S1—O2	119.6 (2)
C7—C8—C13	122.4 (4)	O1—S1—N1	108.7 (2)
C10—C9—C8	123.0 (5)	O2—S1—N1	105.2 (2)
C10—C9—H9	118.5	O1—S1—C1	109.6 (2)
C8—C9—H9	118.5	O2—S1—C1	106.4 (2)
C9—C10—C11	117.8 (5)	N1—S1—C1	106.6 (2)
C9—C10—C14	121.0 (5)		
			
C6—C1—C2—C3	0.7 (6)	C8—C9—C10—C11	−1.2 (7)
S1—C1—C2—C3	−175.5 (3)	C8—C9—C10—C14	179.7 (5)
C6—C1—C2—Cl1	179.9 (3)	C9—C10—C11—C12	0.8 (7)
S1—C1—C2—Cl1	3.7 (6)	C14—C10—C11—C12	179.9 (5)
C1—C2—C3—C4	−0.6 (7)	C10—C11—C12—C7	0.4 (7)
Cl1—C2—C3—C4	−179.9 (4)	C8—C7—C12—C11	−1.2 (7)
C2—C3—C4—C5	0.2 (7)	N1—C7—C12—C11	−178.6 (4)
C2—C3—C4—Cl2	179.6 (4)	C12—C7—N1—S1	−76.9 (5)
C3—C4—C5—C6	0.1 (7)	C8—C7—N1—S1	105.7 (4)
Cl2—C4—C5—C6	−179.3 (4)	C7—N1—S1—O1	46.7 (4)
C4—C5—C6—C1	0.0 (7)	C7—N1—S1—O2	176.0 (3)
C2—C1—C6—C5	−0.4 (7)	C7—N1—S1—C1	−71.4 (4)
S1—C1—C6—C5	176.2 (4)	C2—C1—S1—O1	−32.1 (5)
C12—C7—C8—C9	0.7 (7)	C6—C1—S1—O1	151.6 (3)
N1—C7—C8—C9	178.0 (4)	C2—C1—S1—O2	−162.7 (4)
C12—C7—C8—C13	−178.4 (4)	C6—C1—S1—O2	21.0 (4)
N1—C7—C8—C13	−1.1 (7)	C2—C1—S1—N1	85.5 (4)
C7—C8—C9—C10	0.5 (7)	C6—C1—S1—N1	−90.9 (4)
C13—C8—C9—C10	179.7 (5)		

### Hydrogen-bond geometry (Å, °)

**Table:**

*D*—H···*A*	*D*—H	H···*A*	*D*···*A*	*D*—H···*A*
N1—H1N···O2^i^	0.86 (2)	2.23 (2)	3.059 (5)	164 (4)

Symmetry codes: (i) −*x*, −*y*, −*z*+1.
